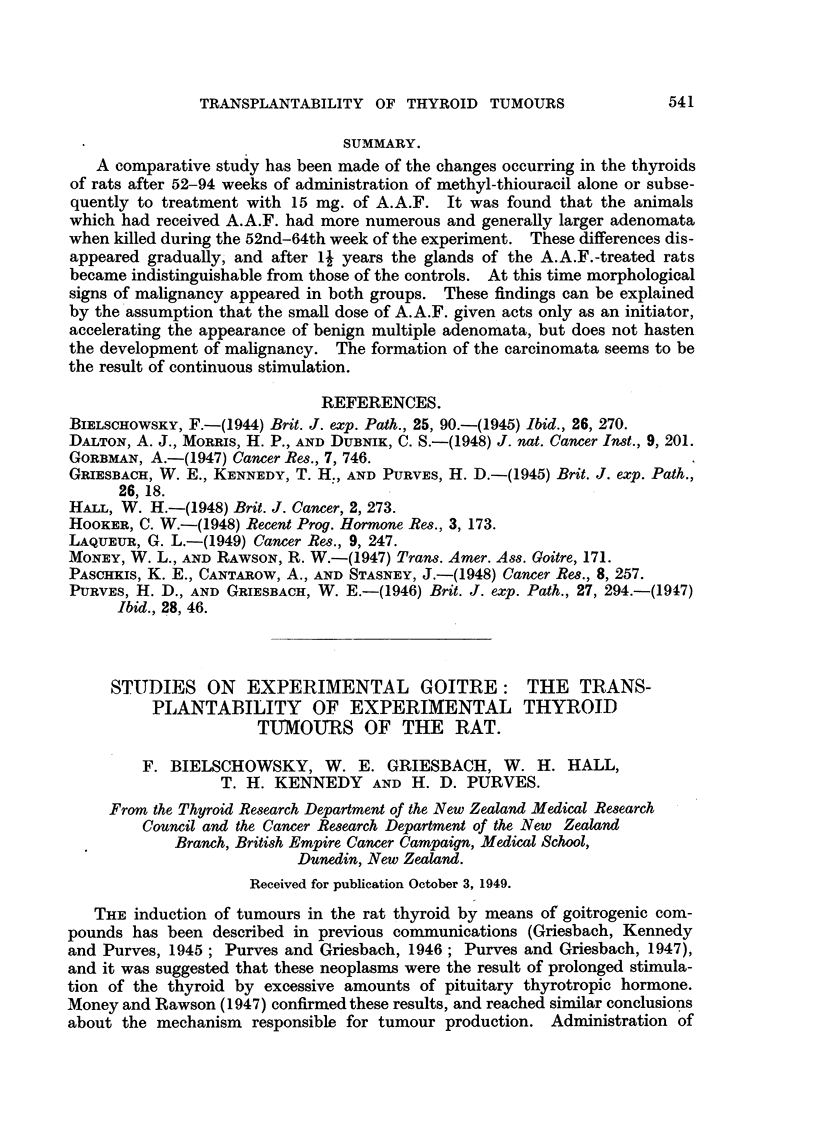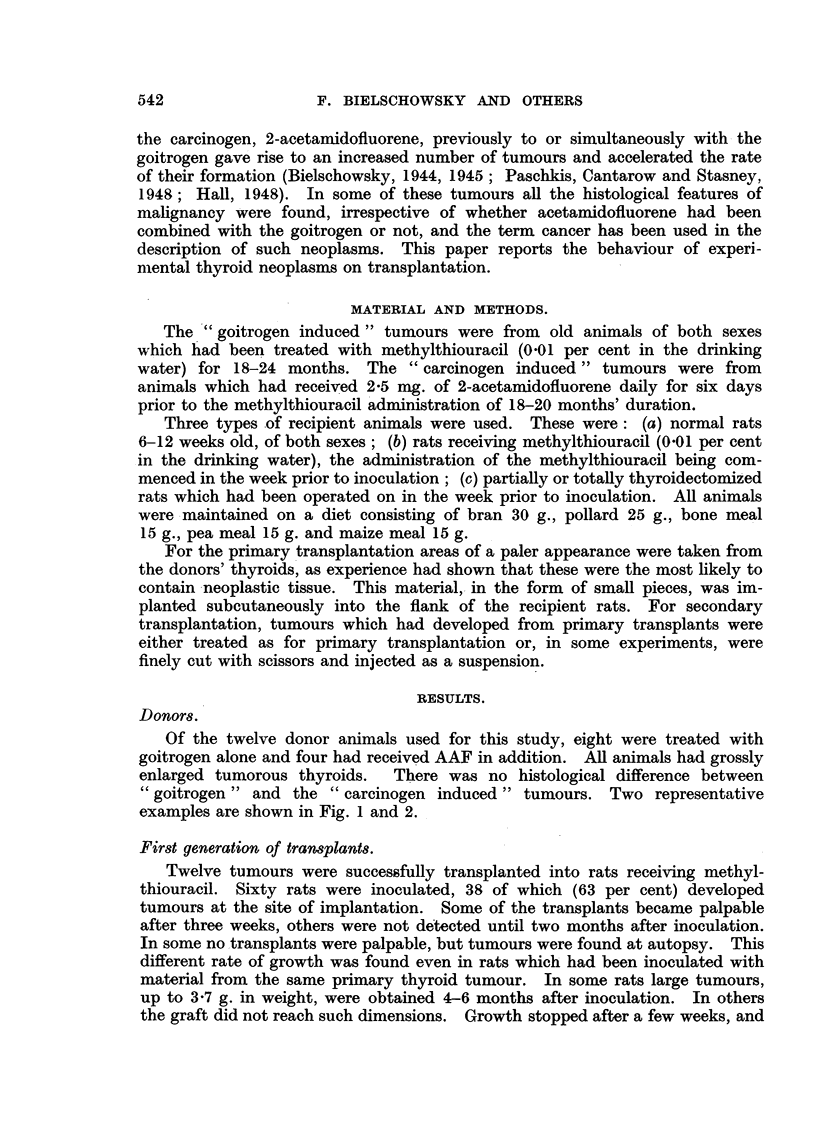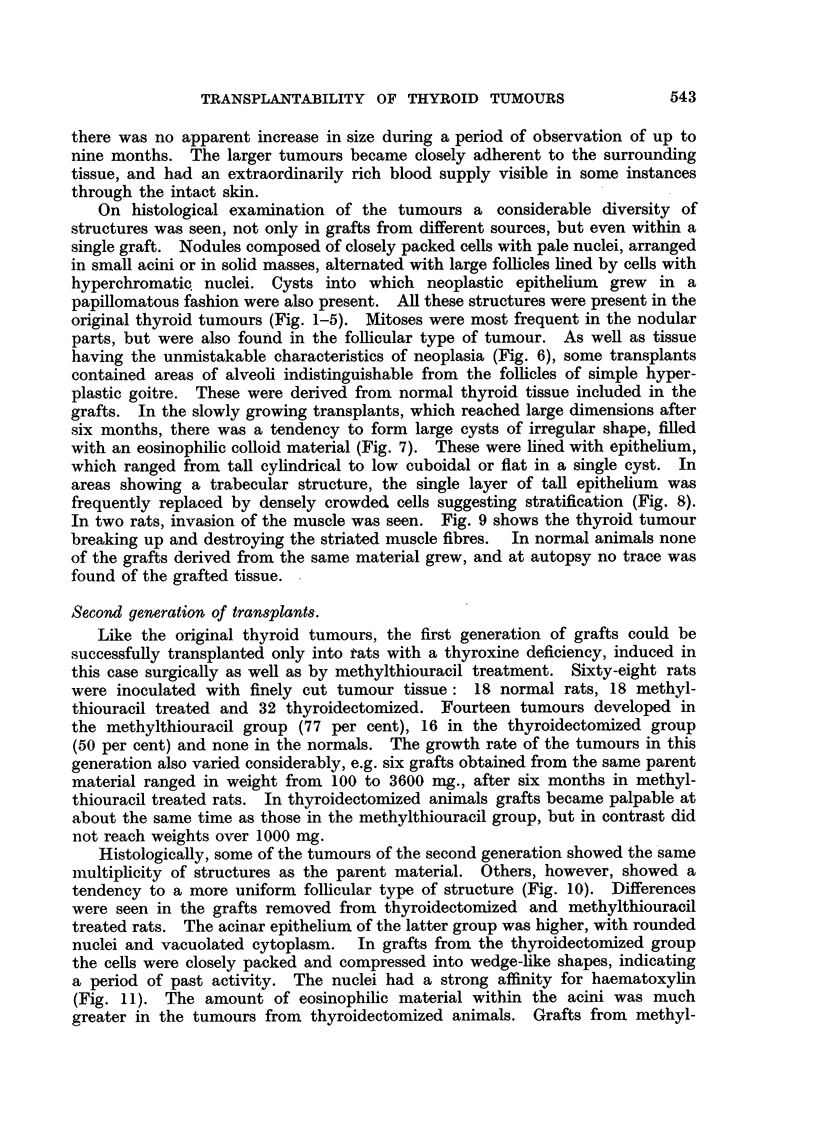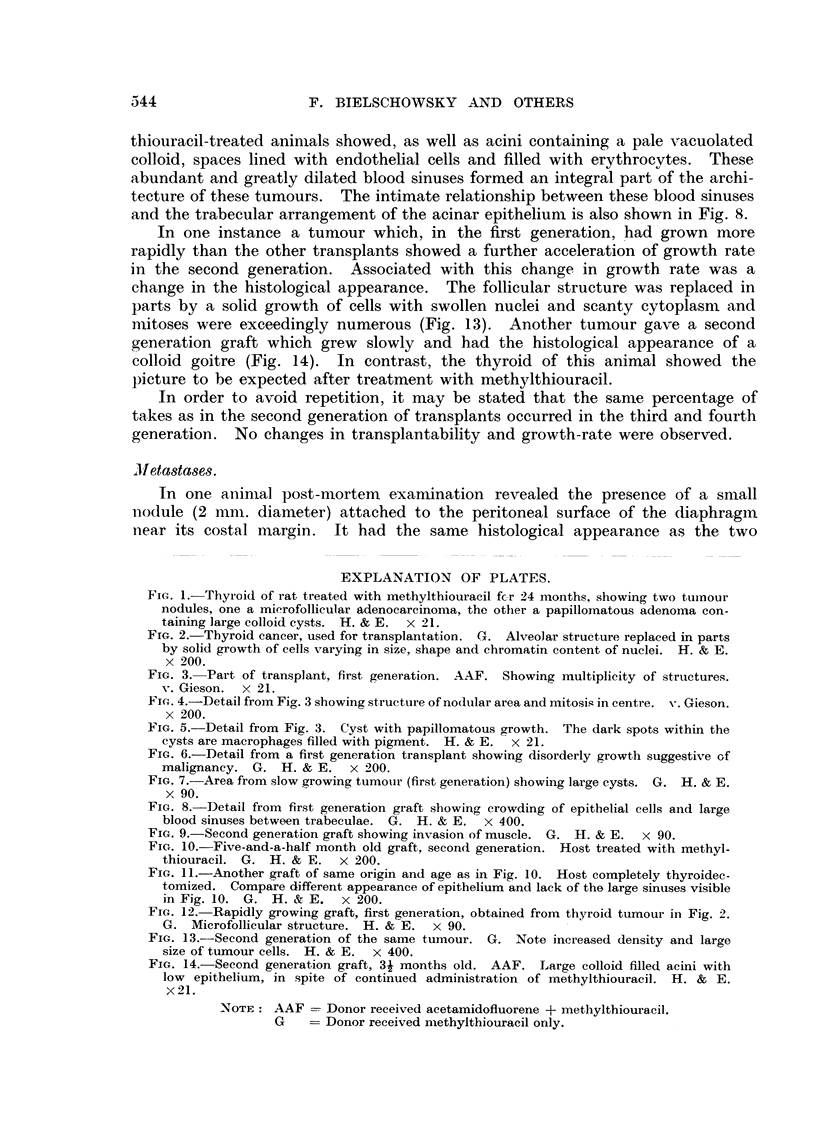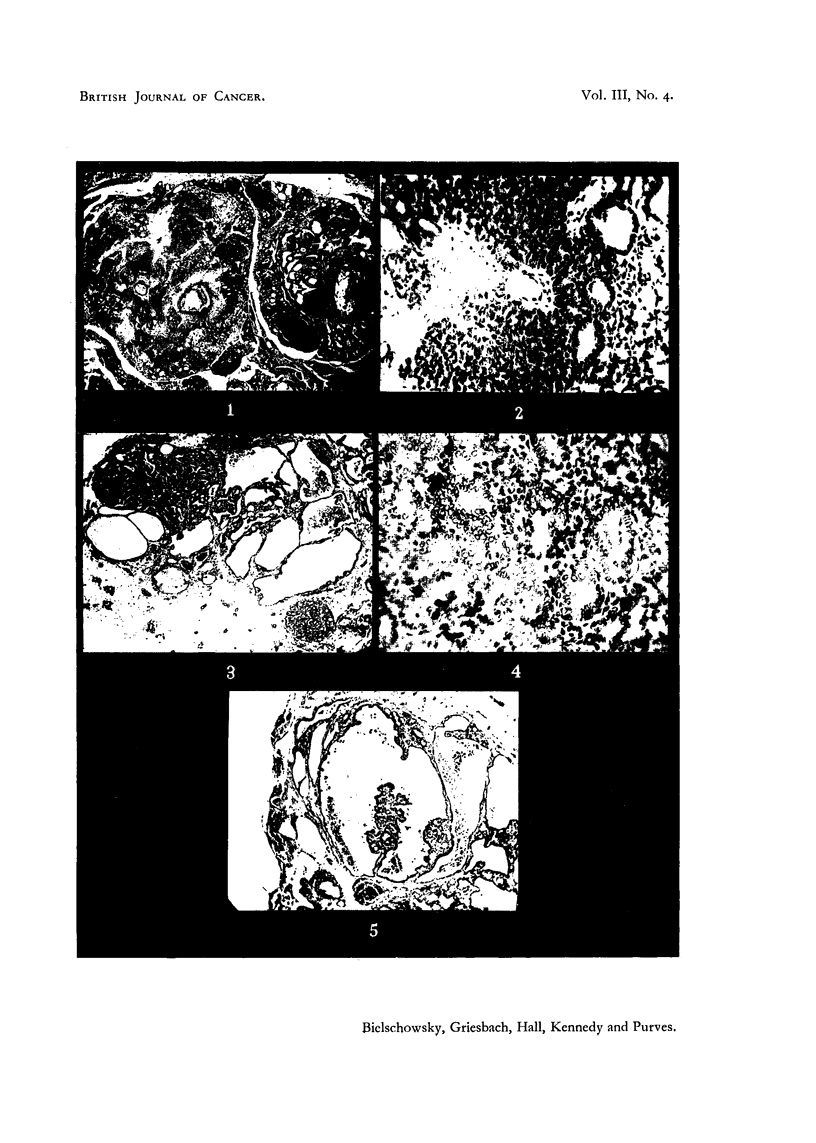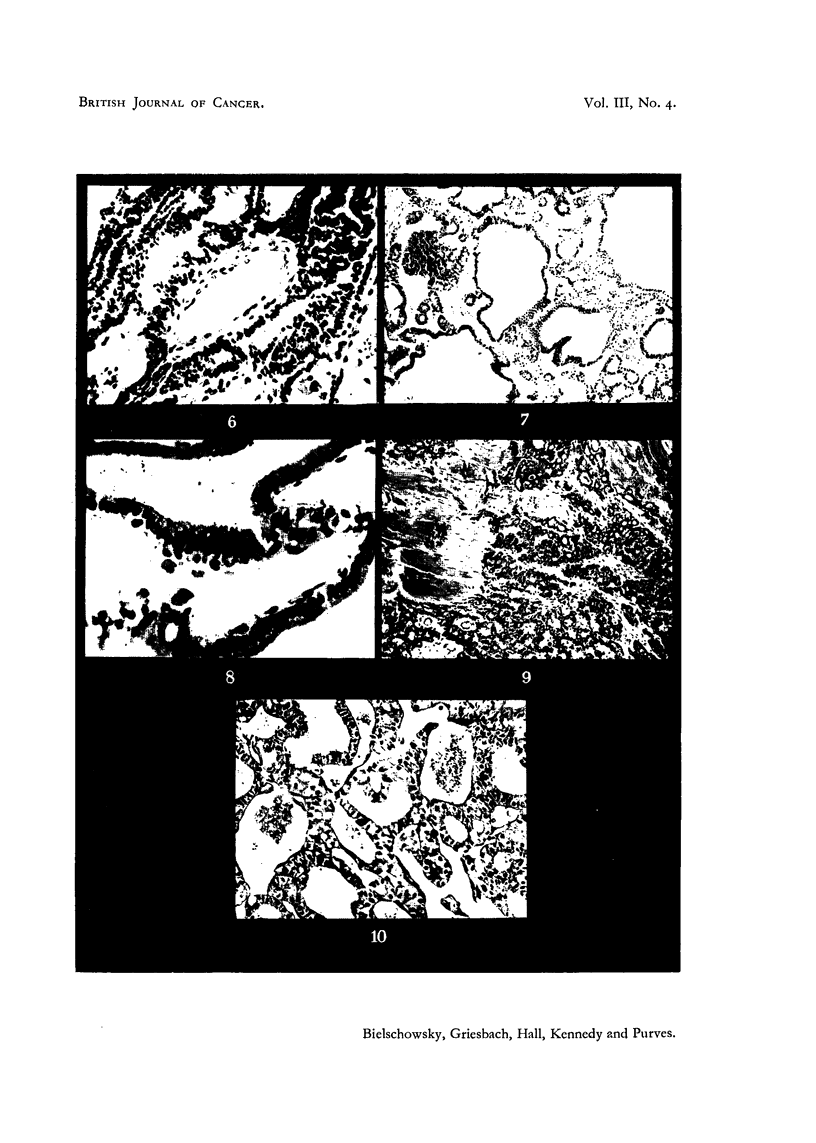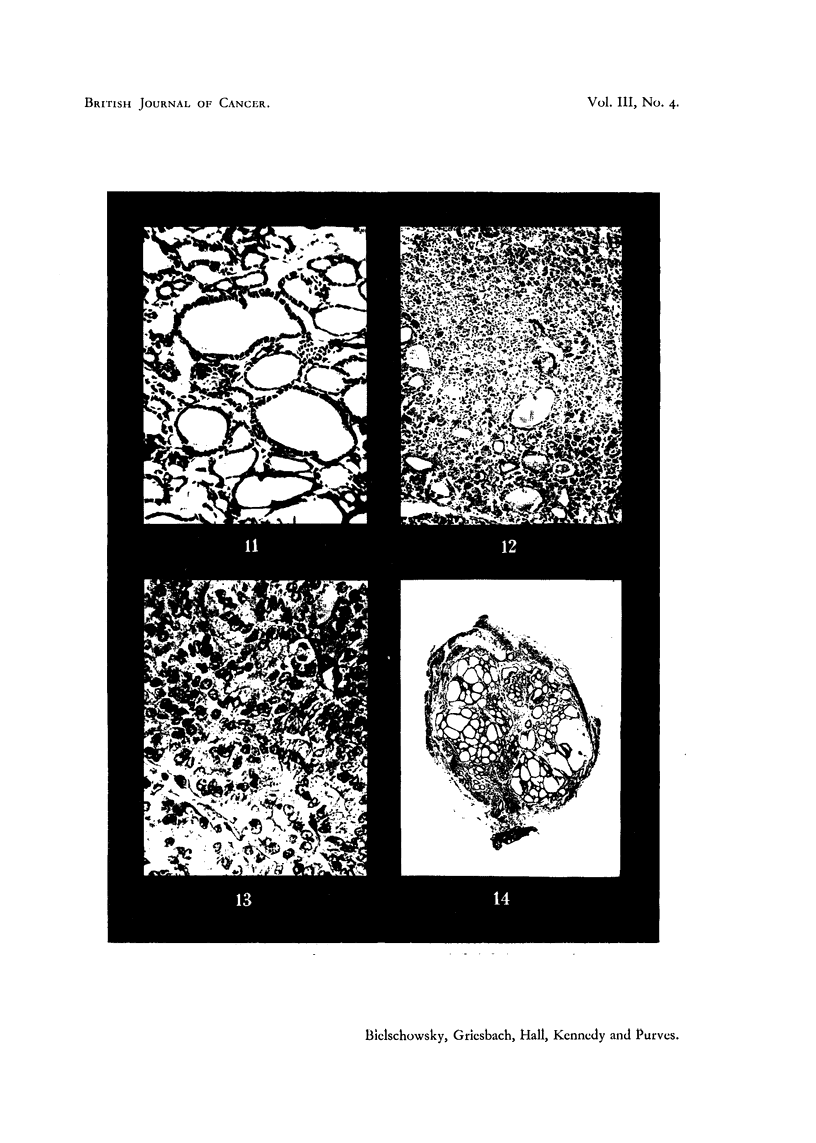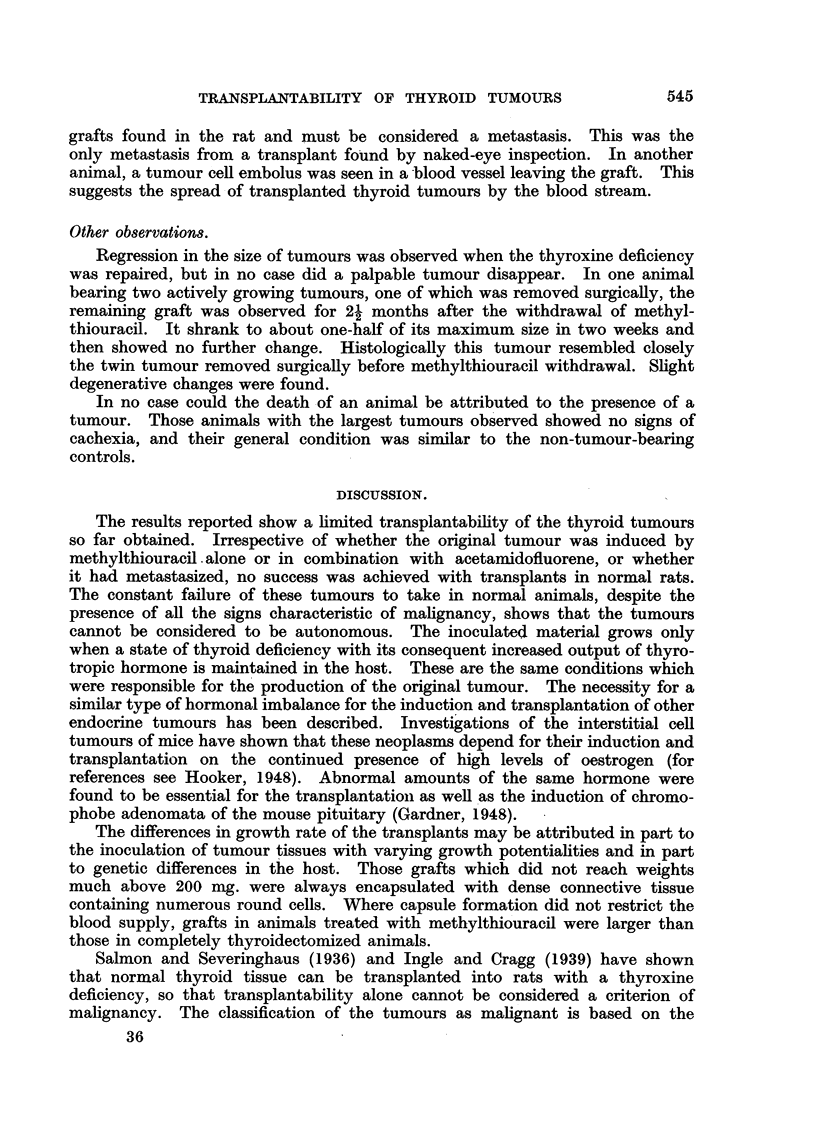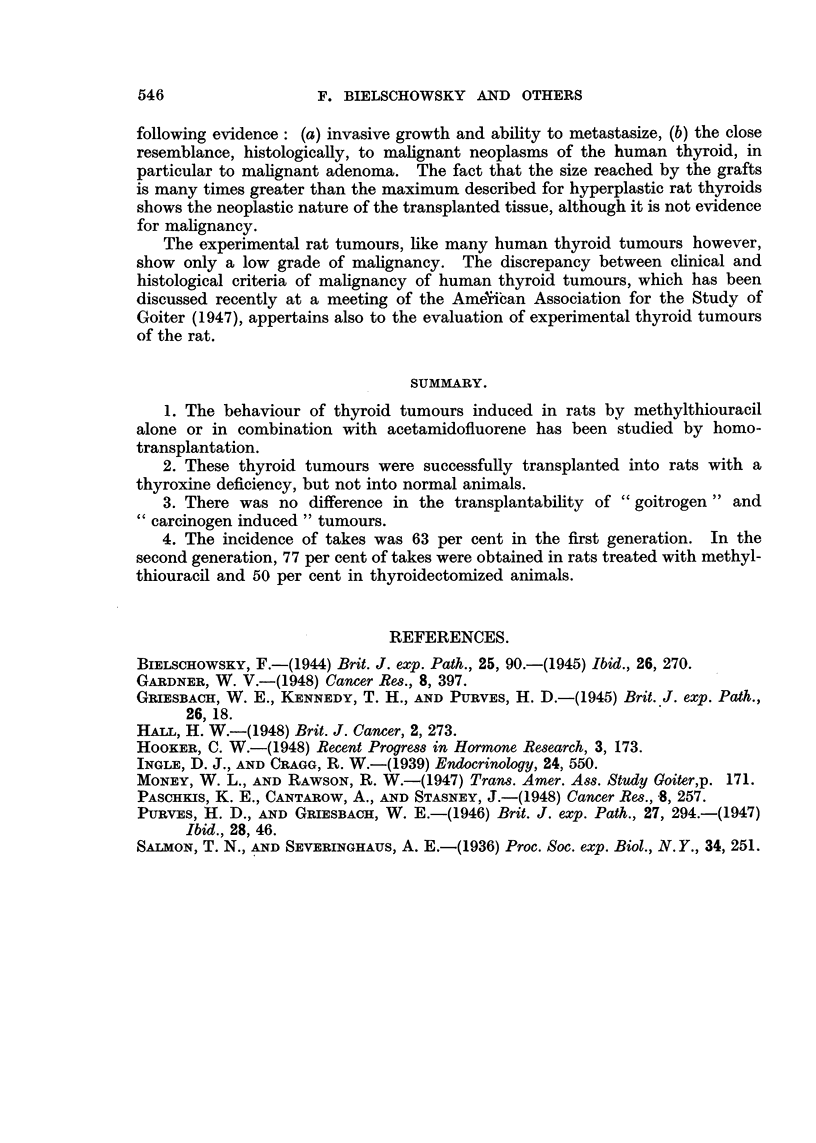# Studies on Experimental Goitre: The Transplantability of Experimental Thyroid Tumours of the Rat

**DOI:** 10.1038/bjc.1949.58

**Published:** 1949-12

**Authors:** F. Bielschowsky, W. E. Griesbach, W. H. Hall, T. H. Kennedy, H. D. Purves

## Abstract

**Images:**


SUMMARY.

### REFERENCES.

Bielschowsky, F.—(1944) *Brit. J. exp. Path.*, **25**, 90.—(1945) *Ibid.*, **26**, 270.

Dalton, A. J., Morris, H. P., and Dubnik, C. S.—(1948) *J. nat. Cancer Inst.*, **9**, 201.

Gorbman, A.—(1947) *Cancer Res.*, **7**, 746.

Griesbach, W. E., Kennedy, T. H., and Purves, H. D.—(1945) *Brit. J. exp. Path.*, **26**, 18.

Hall, W. H.—(1948) *Brit. J. Cancer*, **2**, 273.

Hooker, C. W.—(1948) *Recent Prog. Hormone Res.*, **3**, 173.

Laqueur, G. L.—(1949) *Cancer Res.*, **9**, 247.

Money, W. L., and Rawson, R. W.—(1947) *Trans. Amer. Ass. Goitre*, 171.

Paschkis, K. E., Cantarow, A., and Stasney, J.—(1948) *Cancer Res.*, **8**, 257.

Purves, H. D., and Griesbach, W. E.—(1946) *Brit. J. exp. Path.*, **27**, 294.—(1947) *Ibid.*, **28**, 46.

---

### STUDIES ON EXPERIMENTAL GOITRE: THE TRANSPLANTABILITY OF EXPERIMENTAL THYROID TUMOURS OF THE RAT.

F. BIELSCHOWSKY, W. E. GRIESBACH, W. H. HALL, T. H. KENNEDY and H. D. PURVES.

*From the Thyroid Research Department of the New Zealand Medical Research Council and the Cancer Research Department of the New Zealand Branch, British Empire Cancer Campaign, Medical School, Dunedin, New Zealand.*

Received for publication October 3, 1949.

The induction of tumours in the rat thyroid by means of goitrogenic compounds has been described in previous communications (Griesbach, Kennedy and Purves, 1945; Purves and Griesbach, 1946; Purves and Griesbach, 1947), and it was suggested that these neoplasms were the result of prolonged stimulation of the thyroid by excessive amounts of pituitary thyrotropic hormone. Money and Rawson (1947) confirmed these results, and reached similar conclusions about the mechanism responsible for tumour production. Administration of the carcinogen, 2-acetamidofluorene, previously to or simultaneously with the goitrogen gave rise to an increased number of tumours and accelerated the rate of their formation (Bielschowsky, 1944, 1945; Paschkis, Cantarow and Stasney, 1948; Hall, 1948). In some of these tumours all the histological features of malignancy were found, irrespective of whether acetamidofluorene had been combined with the goitrogen or not, and the term cancer has been used in the description of such neoplasms. This paper reports the behaviour of experimental thyroid neoplasms on transplantation.

### MATERIAL AND METHODS.

The "goitrogen induced" tumours were from old animals of both sexes which had been treated with methylthiouracil (0·01 per cent in the drinking water) for 18–24 months. The "carcinogen induced" tumours were from animals which had received 2·5 mg. of 2-acetamidofluorene daily for six days prior to the methylthiouracil administration of 18–20 months' duration.

Three types of recipient animals were used. These were: (*a*) normal rats 6–12 weeks old, of both sexes; (*b*) rats receiving methylthiouracil (0·01 per cent in the drinking water), the administration of the methylthiouracil being commenced in the week prior to inoculation; (*c*) partially or totally thyroidectomized rats which had been operated on in the week prior to inoculation. All animals were maintained on a diet consisting of bran 30 g., pollard 25 g., bone meal 15 g., pea meal 15 g. and maize meal 15 g.

For the primary transplantation areas of a paler appearance were taken from the donors' thyroids, as experience had shown that these were the most likely to contain neoplastic tissue. This material, in the form of small pieces, was implanted subcutaneously into the flank of the recipient rats. For secondary transplantation, tumours which had developed from primary transplants were either treated as for primary transplantation or, in some experiments, were finely cut with scissors and injected as a suspension.

### RESULTS.

### *Donors.*

Of the twelve donor animals used for this study, eight were treated with goitrogen alone and four had received AAF in addition. All animals had grossly enlarged tumorous thyroids. There was no histological difference between "goitrogen" and the "carcinogen induced" tumours. Two representative examples are shown in Fig. 1 and 2.

### *First generation of transplants.*

Twelve tumours were successfully transplanted into rats receiving methylthiouracil. Sixty rats were inoculated, 38 of which (63 per cent) developed tumours at the site of implantation. Some of the transplants became palpable after three weeks, others were not detected until two months after inoculation. In some no transplants were palpable, but tumours were found at autopsy. This different rate of growth was found even in rats which had been inoculated with material from the same primary thyroid tumour. In some rats large tumours, up to 3·7 g. in weight, were obtained 4–6 months after inoculation. In others the graft did not reach such dimensions. Growth stopped after a few weeks, and there was no apparent increase in size during a period of observation of up to nine months. The larger tumours became closely adherent to the surrounding tissue, and had an extraordinarily rich blood supply visible in some instances through the intact skin.

On histological examination of the tumours a considerable diversity of structures was seen, not only in grafts from different sources, but even within a single graft. Nodules composed of closely packed cells with pale nuclei, arranged in small acini or in solid masses, alternated with large follicles lined by cells with hyperchromatic nuclei. Cysts into which neoplastic epithelium grew in a papillomatous fashion were also present. All these structures were present in the original thyroid tumours (Fig. 1–5). Mitoses were most frequent in the nodular parts, but were also found in the follicular type of tumour. As well as tissue having the unmistakable characteristics of neoplasia (Fig. 6), some transplants contained areas of alveoli indistinguishable from the follicles of simple hyperplastic goitre. These were derived from normal thyroid tissue included in the grafts. In the slowly growing transplants, which reached large dimensions after six months, there was a tendency to form large cysts of irregular shape, filled with an eosinophilic colloid material (Fig. 7). These were lined with epithelium, which ranged from tall cylindrical to low cuboidal or flat in a single cyst. In areas showing a trabecular structure, the single layer of tall epithelium was frequently replaced by densely crowded cells suggesting stratification (Fig. 8). In two rats, invasion of the muscle was seen. Fig. 9 shows the thyroid tumour breaking up and destroying the striated muscle fibres. In normal animals none of the grafts derived from the same material grew, and at autopsy no trace was found of the grafted tissue.

*Second generation of transplants.*

Like the original thyroid tumours, the first generation of grafts could be successfully transplanted only into rats with a thyroxine deficiency, induced in this case surgically as well as by methylthiouracil treatment. Sixty-eight rats were inoculated with finely cut tumour tissue: 18 normal rats, 18 methylthiouracil treated and 32 thyroidectomized. Fourteen tumours developed in the methylthiouracil group (77 per cent), 16 in the thyroidectomized group (50 per cent) and none in the normals. The growth rate of the tumours in this generation also varied considerably, e.g. six grafts obtained from the same parent material ranged in weight from 100 to 3600 mg., after six months in methylthiouracil treated rats. In thyroidectomized animals grafts became palpable at about the same time as those in the methylthiouracil group, but in contrast did not reach weights over 1000 mg.

Histologically, some of the tumours of the second generation showed the same multiplicity of structures as the parent material. Others, however, showed a tendency to a more uniform follicular type of structure (Fig. 10). Differences were seen in the grafts removed from thyroidectomized and methylthiouracil treated rats. The acinar epithelium of the latter group was higher, with rounded nuclei and vacuolated cytoplasm. In grafts from the thyroidectomized group the cells were closely packed and compressed into wedge-like shapes, indicating a period of past activity. The nuclei had a strong affinity for haematoxylin (Fig. 11). The amount of eosinophilic material within the acini was much greater in the tumours from thyroidectomized animals. Grafts from methylthiouracil-treated animals showed, as well as acini containing a pale vacuolated colloid, spaces lined with endothelial cells and filled with erythrocytes. These abundant and greatly dilated blood sinuses formed an integral part of the architecture of these tumours. The intimate relationship between these blood sinuses and the trabecular arrangement of the acinar epithelium is also shown in Fig. 8.

In one instance a tumour which, in the first generation, had grown more rapidly than the other transplants showed a further acceleration of growth rate in the second generation. Associated with this change in growth rate was a change in the histological appearance. The follicular structure was replaced in parts by a solid growth of cells with swollen nuclei and scanty cytoplasm and mitoses were exceedingly numerous (Fig. 13). Another tumour gave a second generation graft which grew slowly and had the histological appearance of a colloid goitre (Fig. 14). In contrast, the thyroid of this animal showed the picture to be expected after treatment with methylthiouracil.

In order to avoid repetition, it may be stated that the same percentage of takes as in the second generation of transplants occurred in the third and fourth generation. No changes in transplantability and growth-rate were observed.

*Metastases.*

In one animal post-mortem examination revealed the presence of a small nodule (2 mm. diameter) attached to the peritoneal surface of the diaphragm near its costal margin. It had the same histological appearance as the two

---

EXPLANATION OF PLATES.

Fig. 1.—Thyroid of rat treated with methylthiouracil for 24 months, showing two tumour nodules, one a microfollicular adenocarcinoma, the other a papillomatous adenoma containing large colloid cysts. H. & E. × 21.

Fig. 2.—Thyroid cancer, used for transplantation. G. Alveolar structure replaced in parts by solid growth of cells varying in size, shape and chromatin content of nuclei. H. & E. × 200.

Fig. 3.—Part of transplant, first generation. AAF. Showing multiplicity of structures. v. Gieson. × 21.

Fig. 4.—Detail from Fig. 3 showing structure of nodular area and mitosis in centre. v. Gieson. × 200.

Fig. 5.—Detail from Fig. 3. Cyst with papillomatous growth. The dark spots within the cysts are macrophages filled with pigment. H. & E. × 21.

Fig. 6.—Detail from a first generation transplant showing disorderly growth suggestive of malignancy. G. H. & E. × 200.

Fig. 7.—Area from slow growing tumour (first generation) showing large cysts. G. H. & E. × 90.

Fig. 8.—Detail from first generation graft showing crowding of epithelial cells and large blood sinuses between trabeculae. G. H. & E. × 400.

Fig. 9.—Second generation graft showing invasion of muscle. G. H. & E. × 90.

Fig. 10.—Five-and-a-half month old graft, second generation. Host treated with methylthiouracil. G. H. & E. × 200.

Fig. 11.—Another graft of same origin and age as in Fig. 10. Host completely thyroidectomized. Compare different appearance of epithelium and lack of the large sinuses visible in Fig. 10. G. H. & E. × 200.

Fig. 12.—Rapidly growing graft, first generation, obtained from thyroid tumour in Fig. 2. G. Microfollicular structure. H. & E. × 90.

Fig. 13.—Second generation of the same tumour. G. Note increased density and large size of tumour cells. H. & E. × 400.

Fig. 14.—Second generation graft, 3½ months old. AAF. Large colloid filled acini with low epithelium, in spite of continued administration of methylthiouracil. H. & E. ×21.

Note : AAF = Donor received acetamidofluorene + methylthiouracil.
G = Donor received methylthiouracil only.

1

2

3

4

5

6 7 8 9 10

Bielschowsky, Griesbach, Hall, Kennedy and Purves.

11

12

13

14

Bielschowsky, Griesbach, Hall, Kennedy and Purves.

grafts found in the rat and must be considered a metastasis. This was the only metastasis from a transplant found by naked-eye inspection. In another animal, a tumour cell embolus was seen in a blood vessel leaving the graft. This suggests the spread of transplanted thyroid tumours by the blood stream.

*Other observations.*

Regression in the size of tumours was observed when the thyroxine deficiency was repaired, but in no case did a palpable tumour disappear. In one animal bearing two actively growing tumours, one of which was removed surgically, the remaining graft was observed for 2½ months after the withdrawal of methylthiouracil. It shrank to about one-half of its maximum size in two weeks and then showed no further change. Histologically this tumour resembled closely the twin tumour removed surgically before methylthiouracil withdrawal. Slight degenerative changes were found.

In no case could the death of an animal be attributed to the presence of a tumour. Those animals with the largest tumours observed showed no signs of cachexia, and their general condition was similar to the non-tumour-bearing controls.

### DISCUSSION.

The results reported show a limited transplantability of the thyroid tumours so far obtained. Irrespective of whether the original tumour was induced by methylthiouracil alone or in combination with acetamidofluorene, or whether it had metastasized, no success was achieved with transplants in normal rats. The constant failure of these tumours to take in normal animals, despite the presence of all the signs characteristic of malignancy, shows that the tumours cannot be considered to be autonomous. The inoculated material grows only when a state of thyroid deficiency with its consequent increased output of thyrotropic hormone is maintained in the host. These are the same conditions which were responsible for the production of the original tumour. The necessity for a similar type of hormonal imbalance for the induction and transplantation of other endocrine tumours has been described. Investigations of the interstitial cell tumours of mice have shown that these neoplasms depend for their induction and transplantation on the continued presence of high levels of oestrogen (for references see Hooker, 1948). Abnormal amounts of the same hormone were found to be essential for the transplantation as well as the induction of chromophobe adenomata of the mouse pituitary (Gardner, 1948).

The differences in growth rate of the transplants may be attributed in part to the inoculation of tumour tissues with varying growth potentialities and in part to genetic differences in the host. Those grafts which did not reach weights much above 200 mg. were always encapsulated with dense connective tissue containing numerous round cells. Where capsule formation did not restrict the blood supply, grafts in animals treated with methylthiouracil were larger than those in completely thyroidectomized animals.

Salmon and Severinghaus (1936) and Ingle and Cragg (1939) have shown that normal thyroid tissue can be transplanted into rats with a thyroxine deficiency, so that transplantability alone cannot be considered a criterion of malignancy. The classification of the tumours as malignant is based on the following evidence : (*a*) invasive growth and ability to metastasize, (*b*) the close resemblance, histologically, to malignant neoplasms of the human thyroid, in particular to malignant adenoma. The fact that the size reached by the grafts is many times greater than the maximum described for hyperplastic rat thyroids shows the neoplastic nature of the transplanted tissue, although it is not evidence for malignancy.

The experimental rat tumours, like many human thyroid tumours however, show only a low grade of malignancy. The discrepancy between clinical and histological criteria of malignancy of human thyroid tumours, which has been discussed recently at a meeting of the American Association for the Study of Goiter (1947), appertains also to the evaluation of experimental thyroid tumours of the rat.

### SUMMARY.

1. The behaviour of thyroid tumours induced in rats by methylthiouracil alone or in combination with acetamidofluorene has been studied by homotransplantation.

2. These thyroid tumours were successfully transplanted into rats with a thyroxine deficiency, but not into normal animals.

3. There was no difference in the transplantability of " goitrogen " and " carcinogen induced " tumours.

4. The incidence of takes was 63 per cent in the first generation. In the second generation, 77 per cent of takes were obtained in rats treated with methylthiouracil and 50 per cent in thyroidectomized animals.

### REFERENCES.

Bielschowsky, F.—(1944) *Brit. J. exp. Path.*, **25**, 90.—(1945) *Ibid.*, **26**, 270.
Gardner, W. V.—(1948) *Cancer Res.*, **8**, 397.
Griesbach, W. E., Kennedy, T. H., and Purves, H. D.—(1945) *Brit. J. exp. Path.*, **26**, 18.
Hall, H. W.—(1948) *Brit. J. Cancer*, **2**, 273.
Hooker, C. W.—(1948) *Recent Progress in Hormone Research*, **3**, 173.
Ingle, D. J., and Cragg, R. W.—(1939) *Endocrinology*, **24**, 550.
Money, W. L., and Rawson, R. W.—(1947) *Trans. Amer. Ass. Study Goiter*,p. 171.
Paschkis, K. E., Cantarow, A., and Stasney, J.—(1948) *Cancer Res.*, **8**, 257.
Purves, H. D., and Griesbach, W. E.—(1946) *Brit. J. exp. Path.*, **27**, 294.—(1947) *Ibid.*, **28**, 46.
Salmon, T. N., and Severinghaus, A. E.—(1936) *Proc. Soc. exp. Biol., N.Y.*, **34**, 251.